# Magnetoencephalography Brain Signatures Relate to Cognition and Cognitive Reserve in the Oldest-Old: The EMIF-AD 90 + Study

**DOI:** 10.3389/fnagi.2021.746373

**Published:** 2021-11-25

**Authors:** Alessandra Griffa, Nienke Legdeur, Maryam Badissi, Martijn P. van den Heuvel, Cornelis J. Stam, Pieter Jelle Visser, Arjan Hillebrand

**Affiliations:** ^1^Division of Neurology, Department of Clinical Neurosciences, Geneva University Hospitals and Faculty of Medicine, University of Geneva, Geneva, Switzerland; ^2^Center of Neuroprosthetics, Institute of Bioengineering, École Polytechnique Fédérale De Lausanne (EPFL), Geneva, Switzerland; ^3^Department of Clinical Neurophysiology and MEG Center, Amsterdam Neuroscience, Amsterdam UMC, Vrije Universiteit Amsterdam, Amsterdam, Netherlands; ^4^Department of Neurology, Amsterdam Neuroscience, Alzheimer Center Amsterdam, Vrije Universiteit Amsterdam, Amsterdam UMC, Amsterdam, Netherlands; ^5^Dutch Connectome Lab, Department of Complex Trait Genetics, Center for Neuroscience and Cognitive Research, Amsterdam Neuroscience, Vrije Universiteit Amsterdam, Amsterdam UMC, Amsterdam, Netherlands; ^6^Department of Psychiatry and Neuropsychology, School for Mental Health and Neuroscience, Maastricht University, Maastricht, Netherlands

**Keywords:** cognition, functional connectivity, cognitive reserve, oldest-old, magnetoencephalography

## Abstract

The oldest-old subjects represent the fastest growing segment of society and are at high risk for dementia with a prevalence of up to 40%. Lifestyle factors, such as lifelong participation in cognitive and leisure activities, may contribute to individual cognitive reserve and reduce the risk for cognitive impairments. However, the neural bases underlying cognitive functioning and cognitive reserve in this age range are still poorly understood. Here, we investigate spectral and functional connectivity features obtained from resting-state MEG recordings in a cohort of 35 cognitively normal (92.2 ± 1.8 years old, 19 women) and 11 cognitively impaired (90.9 ± 1.9 years old, 1 woman) oldest-old participants, in relation to cognitive traits and cognitive reserve. The latter was approximated with a self-reported scale on lifelong engagement in cognitively demanding activities. Cognitively impaired oldest-old participants had slower cortical rhythms in frontal, parietal and default mode network regions compared to the cognitively normal subjects. These alterations mainly concerned the theta and beta band and partially explained inter-subject variability of episodic memory scores. Moreover, a distinct spectral pattern characterized by higher relative power in the alpha band was specifically associated with higher cognitive reserve while taking into account the effect of age and education level. Finally, stronger functional connectivity in the alpha and beta band were weakly associated with better cognitive performances in the whole group of subjects, although functional connectivity effects were less prominent than the spectral ones. Our results shed new light on the neural underpinnings of cognitive functioning in the oldest-old population and indicate that cognitive performance and cognitive reserve may have distinct spectral electrophysiological substrates.

## Introduction

The oldest-old population, including individuals aged 85–90 years and older, is the fastest growing segment of Western societies (Corrada et al., 2010; Legdeur et al., 2018). The number of oldest-old is estimated to increase fivefold in the coming decades, resulting in 77 millions of oldest-old individuals worldwide by 2050 (United Nations, Department of Economic and Social Affairs, Population Division, 2019). Many of these individuals will suffer from cognitive impairments and dementia, with a dementia prevalence of up to 40% in this age range and major implications for public health and society (Bullain and Corrada, 2013; Yang et al., 2013). The identification of dementia’s neuropathological substrate becomes increasingly challenging with age (Yang et al., 2013). This is due to an increasing prevalence of Alzheimer’s and cerebrovascular pathologies (the most common causes of dementia) among non-demented oldest-old individuals (Wharton et al., 2011; Paolacci et al., 2017; Legdeur et al., 2019), and to a more frequent co-occurrence of multiple dementia-related pathologies (Corrada et al., 2012; James et al., 2012). In parallel, convergent evidence suggests that different lifestyle factors may contribute to individual cognitive reserve-defined as the adaptability of functional brain processes to cope with aging or pathological processes (Stern, 2009; Stern et al., 2018)- and protect from, or delay cognitive decline and incidence of clinical dementia (Verghese et al., 2003; Pettigrew et al., 2019; Soldan et al., 2021) even in presence of extensive brain pathologies (Xu et al., 2019). Yet, the neural underpinnings of cognitive functioning and history of lifelong engagement in cognitive activities in the oldest-old population are not clear.

Few electrophysiological studies have investigated the brain functional substrate of cognitive impairments in the oldest-old population, since data for this age range are scarce (Yang et al., 2013; Legdeur et al., 2018). Studies that used electroencephalography (EEG) or magnetoencephalography (MEG) in older adults aged 65–80 years found that demented subjects and subjects at risk of developing dementia have brain functional alterations with slowing of cortical oscillations (Babiloni et al., 2006; Fernández et al., 2006; van der Hiele et al., 2007; de Haan et al., 2008), reduced functional connectivity in the higher frequency bands in posterior, parietal and limbic brain regions, and stronger functional connectivity between frontal and posterior areas (Engels et al., 2017; Miraglia et al., 2017; Maestú et al., 2019; Babiloni et al., 2020a). However, it is unknown whether comparable spectral and functional connectivity patterns are observable in cognitively impaired oldest-old compared to cognitively normal oldest-old, and how these patterns could relate to protective and cognitive reserve factors.

Little research has been done on the electrophysiological substrate of cognitive reserve (Šneidere et al., 2020; Balart-Sánchez et al., 2021). Results on resting-state data are controversial with studies reporting involvement of alpha rhythms (Babiloni et al., 2020b), gamma rhythms (Yang and Lin, 2020), or no association with cognitive reserve (López et al., 2014). One study found negative and positive associations between whole-brain EEG functional connectivity and cognitive reserve in younger and older healthy adults, respectively, suggesting possible shifts in the relationship between brain electrophysiology and cognitive reserve with aging (Fleck et al., 2017). In light of these considerations, understanding the possibly age-specific (Gonzalez-Escamilla et al., 2018) neural underpinning of cognitive impairment and cognitive reserve in the oldest-old is key to identifying protective factors for cognitive decline, testing prevention and treatment options, and monitoring dementia-related pathological evolution in this age segment.

MEG is a neuroimaging technique that allows quantifying electrophysiological patterns at the individual subject level by probing the magnetic fields associated with postsynaptic potentials by means of sensor arrays that cover the whole head (Hämäläinen et al., 1993; Stam, 2010; Hari and Puce, 2017; Gross, 2019). Signal contributions from different brain regions can be estimated from sensor-level data using source-reconstruction algorithms (Baillet et al., 2001), including beamforming techniques (Hillebrand et al., 2005), and further analyzed to elucidate spectral features of neuronal activity and functional couplings between regions (Hillebrand et al., 2012). MEG studies have revealed the functional organization of the brain across different frequency bands into large-scale systems, including the visual, sensorimotor and default mode networks (de Pasquale et al., 2010; Brookes et al., 2011; Hipp et al., 2012), and its disruption in neurodegenerative disorders (Stam, 2014) and dementia (Stam, 2010; Engels et al., 2017; Hughes et al., 2019) but have not been applied to the oldest-old. The objective of this study is to elucidate the relation between spectral and functional connectivity properties of MEG oscillations and cognitive impairments in a unique cohort of oldest-old subjects from the EMIF-AD 90 + Study (Legdeur et al., 2018), and to investigate the relationship between these neural biomarkers and lifelong engagement in cognitively demanding activity, a possible protective factor for cognitive decline and proxy for cognitive reserve (Stern, 2009; Landau et al., 2012).

## Materials and Methods

### Subjects

60 subjects (91.8 ± 2.0 years of age, 37 females) were recruited at the Amsterdam University Medical Centers (Amsterdam UMC), The Netherlands, in the framework of the EMIF-AD (European Medical Information Framework for AD) 90 + Study (Legdeur et al., 2018), a case-control study with cognitively normal and impaired individuals to investigate the protective factors for cognitive impairment in the oldest-old population. In order to increase the power of our study and in agreement with others (Bullain and Corrada, 2013), we also included 4 subjects aged between 88 and 90 years. Neurological disorders (e.g., stroke or epilepsy), severe depression (Geriatric Depression Scale (GDS) > 11) (Yesavage et al., 1982) and visual or auditory impairments that made neuropsychological testing impossible, were exclusion criteria. Moreover, 14 out of 60 subjects were excluded from further analyses because of missing MRI data, low-quality MEG recordings or poor MRI-MEG co-registration (see below), so that a restricted subset of 46 subjects (91.9 ± 1.9 years of age, 29 females) were included in the final analyses. This study was approved by the local Medical Ethics Review Committee of the Amsterdam UMC, and all subjects provided written informed consent.

### Clinical and Cognitive Assessment

Each participant underwent a comprehensive neuropsychological, functional and clinical assessment. Neuropsychological and functional testing was administrated by a neuropsychologist; clinical diagnosis was made by a neurologist, geriatrician or general practitioner (McKhann et al., 1984; Petersen, 2004). Subjects were considered cognitively normal (CN) if they scored 0 points on the Clinical Dementia Rating (CDR) scale (Morris, 1993) and had no clinical diagnosis of dementia or mild cognitive impairment (35 CN, 92.2 ± 1.8 years of age, 19 females). Cognitively Impaired (CI) subjects (11 CI, 90.9 ± 1.9 years of age, 10 females) had a CDR score larger than 0 points (median CDR = 1) and a clinical diagnosis of probable Alzheimer’s disease (AD, 10 subjects) or amnestic mild cognitive impairment (aMCI, 1 subject).

The overall cognitive ability of each participant was assessed with the Mini-Mental State Examination (MMSE) (Folstein et al., 1983). Executive control was tested with the letter fluency test (1 min per letter, letters D-A-T) (Tombaugh et al., 1999), the processing speed with the Trail Making Tests (TMT)-B score (Reitan, 1958; Broshek and Barth, 2000), and episodic memory with the total score of the CERAD (Consortium to Establish a Registry for Alzheimer’s Disease) battery over three trials (Rossetti et al., 2010). Lifelong engagement in cognitive activities was assessed with a retrospective self-reported scale quantifying how often the participant engaged in common cognitively demanding activities that depend minimally on socioeconomic status, such as reading books or newspapers, playing games or writing letters (Wilson et al., 2003; Landau et al., 2012). Specifically, each participant was asked to rate her/his engagement in these activities at 6, 12, 18, 40, and current years of age, according to a 5-level frequency scale (once a year or never/several times a year/several times a month/several times a week/several times a day). From the questionnaire responses, two composite scores were computed: the current cognitive activity (cCAQ) (average score at current age), and the past cognitive activity (pCAQ) (average score across ages 6, 12, 18, and 40 years) (Landau et al., 2012). The lifelong engagement in leisure and cognitively stimulating activities has been associated with lower dementia risk (Verghese et al., 2003; León et al., 2014; Wang et al., 2017), slower hippocampal atrophy (Valenzuela et al., 2008) and amyloid accumulation (Landau et al., 2012) in aging, and it is considered a proxy of individual cognitive reserve.

### Brain Imaging

#### Magnetic Resonance Imaging Acquisition and Processing

Each subject underwent an MRI session on a 3T Philips Achieva scanner equipped with an 8-channel head coil, which included a structural three-dimensional (3D) T1-weighted acquisition (sagittal gradient-echo sequence; isotropic voxel size 1 × 1 × 1 mm^3^, TR 7.9 ms, TE 4.5 ms, flip angle 8°). T1-weighted volumes were skull-stripped, corrected for intensity inhomogeneity, and segmented into gray matter, white matter, and cerebrospinal fluid compartments with the Statistical Parametric Mapping (SPM) toolbox, version 8 (Penny et al., 2011). The gray matter compartment was then parcellated into 78 cortical regions of interest (ROIs) according to the Automatic Anatomical Labeling (AAL) atlas and 2 hippocampal regions (Tzourio-Mazoyer et al., 2002; Gong et al., 2009; Supplementary Table 1) through spatial normalization of the T1-weighted volumes to MNI space and application of the inverse MNI-to-native transform to bring the parcellation volume to native space [SPM version 8 (Penny et al., 2011)]. The correspondence between the 80 gray matter regions and the 7 resting state networks (RSNs) defined by Yeo et al. (2011) was assessed with a majority-voting procedure in MNI space (MNI-normalized atlases from the Lead-DBS database (Horn and Kühn, 2015) were used) using in-house MATLAB code (Supplementary Table 1).

#### Magnetoencephalography Recording and Preprocessing

Magnetic fields were recorded with a 306-channel whole-head MEG system (Elekta Neuromag Oy, Helsinki, Finland) inside a magnetically shielded room (Vacuumschmelze, Hanau, Germany), at a sampling frequency of 1,250 Hz. An online anti-aliasing filter of 410 Hz and a high-pass filter of 0.1 Hz were applied to sensor-level signals. The MEG protocol consisted of a 5-min eyes-closed recording in resting-state condition, during which subjects were instructed to remain awake and cognitively alert, but they were not assigned any specific task.

Sensor-level time-series were visually inspected to identify ‘bad’ channels (i.e., flat channels and channels affected by high-frequency noise or jump artifacts), which were excluded before applying temporal signal-space separation (tSSS) (min/median/max = 6/11/13 excluded channels per subject). Next, artifact components originating from outside the head volume, including both external noise sources and biomagnetic sources, were removed with the tSSS algorithm implemented in MaxFilter software (Elekta Neuromag Oy, version 2.2.15) (Taulu and Simola, 2006; Taulu and Hari, 2009). For the tSSS parameter setting, an automatic adjustment of the subjects’ sphere center coordinates (Supplementary Material SI.1 and Supplementary Figures 1, 2), a subspace correlation limit of 0.9, and a sliding window of 10 s were used.

The position of the head with respect to the MEG sensors was assessed by means of five Head Position Indicator (HPI) coils and monitored during the recording. The outline of each subject’s scalp (approximatively 500 points) and the HPI coils were digitized with a 3D digitizer (Fastrak, Polhemus, Colchester, VT, United States), and registered to the MRI space using a surface-matching procedure with an approximate accuracy of 4 mm (Whalen et al., 2008). A sphere was then fitted to the outline of the scalp as obtained from the co-registered MRI, which was used as a volume conductor model for the beamformer algorithm (see next section).

#### Source Reconstruction

In order to obtain source-localized activity, the sensor-level preprocessed time-series were projected to 80 locations (sources) in the cortex corresponding to the centroids of the AAL and bilateral hippocampal ROIs, using a beamforming approach (Hillebrand et al., 2012, 2016). Briefly, the sensor-level data were spatially filtered to estimate the contribution to each source’s time-series. For each source, the filter weights were determined from the broad-band (0.5–48 Hz) data covariance matrix and the forward solution (lead field) of the target source according to a scalar minimum variance beamformer (Hillebrand and Barnes, 2005; Hillebrand et al., 2005).

From the source-reconstructed time-series, 8 (not necessarily consecutive) epochs of 13.1 s duration (16,384 samples) were selected for each subject using an automatic procedure. Epochs possibly corrupted by artifacts or during which the subjects may have been drowsy were identified and discarded, based on the presence of extreme values in the temporal domain (indicators of artifacts such as eye movement or high frequency noise), individual peak frequency (IPF) outliers, and low alpha1 occipital power content (indicators of transition to the first stages of sleep; Hari and Puce, 2017; Supplementary Material SI.2 and Supplementary Figure 3). Out of the remaining epochs, the 8 epochs with the highest individual alpha peak frequency and alpha1 occipital power content were selected for each subject, in order to include an equal amount of data for each subject while avoiding possible drowsiness biases across subjects (Supplementary Material SI.2 and Supplementary Figure 3). A random subsample of the epochs selected by this automatic procedure was visually inspected to ensure data quality.

#### Spectral Analysis

For each selected epoch (16,384 samples), the power spectral densities (PSDs) of the source-level time-series were estimated using the periodogram method implemented in MATLAB. The IPF was computed as the frequency at which the average PSD in the occipital regions peaked (Supplementary Table 1), in the range 4–13 Hz. The total power (i.e., the integral of the PSD) in the frequency range 0.5–48 Hz, and the relative band power (RBP) in the delta (0.5–4 Hz), theta (4–8 Hz), alpha1 (8–10 Hz), alpha2 (10–13 Hz), beta (13–30 Hz) and gamma (30–48 Hz) band (i.e., the integral of the PSD in each frequency range, normalized by the total power) were computed for each ROI, epoch, and subject. Values were then averaged over epochs in order to obtain single values per ROI per subject.

#### Functional Connectivity Analysis

Single epoch MEG data were used to build 80 × 80 functional connectivity matrices for each frequency band of interest. For each epoch and subject, the source-level time-series were band-pass filtered into the six bands of interest (delta, theta, alpha1, alpha2, beta, and gamma) using a two-way least-square finite impulse response (FIR) filtering as implemented in EEGLAB (Delorme and Makeig, 2004). Band-pass filtered time-series were then pair-wised orthogonalized to correct for the effects of spatial leakage (i.e., removing zero-lag coupling components). This correction scheme was applied at the single epoch level, and in both directions (orthogonalization of a signal *i* with respect to a signal *j*, and *vice versa*). Next, orthogonalized time-series were Hilbert-transformed and their amplitude envelopes (magnitude of the analytic signal) were pair-wise correlated using the Pearson’s correlation coefficient, thus computing the corrected Amplitude Envelop Correlation (AECc) (Brookes et al., 2012; Hipp et al., 2012). The AECc is a robust functional connectivity measure comprised between −1 and 1 that demonstrates high levels of within- and between-subject consistency and group-level reproducibility (Colclough et al., 2016; Sareen et al., 2021). The resulting functional connectivity matrices were then made symmetric by averaging their upper and lower triangular parts, averaged over the 8 epochs, and used to compute (i) the average functional connectivity at the whole-brain level (i.e., the average over all functional connections between the 80 cortical ROIs), and (ii) the nodal functional connectivity strength (i.e., the row-wise sum of the functional connectivity matrices) for each subject. Group-average functional connectivity matrices for the CI and CN group are shown in Supplementary Figure 4.

### Statistical Analyses

Statistical differences between the CI and CN group were assessed with ANCOVA analyses within a general linear model (GLM) formulation. Age and gender were added as covariates in all the analyses. Considering that functional connectivity and band power content are positively related (Demuru et al., 2020), the RBP was added as covariate in supplementary analyses when comparing functional connectivity values. The effect size was quantified with the Cohen’s *d* coefficient (Cohen, 2013) between GLM residual distributions, after correcting for covariates. When multiple comparisons were performed (e.g., when comparing region-wise RBP or functional connectivity strength), the false discovery rate (*FDR*) was controlled at 0.05 level with the Benjamini-Hochberg procedure (Meskaldji et al., 2013). Pair-wise associations between cognitive scores were assessed with the Spearman’s rank correlation coefficient (ρ). Multivariate relationships between spectral or functional connectivity brain features and cognitive scores (including cognitive reserve indicators) were assessed with partial least square correlation (PLSC) analyses (Krishnan et al., 2011). PLSC identifies multivariate correlation patterns through singular value decomposition of the data covariance matrix. This operation results in a set of orthogonal and paired brain and cognitive saliences, each one representing a pattern of brain and cognitive features with maximum covariance. To interpret the brain and cognitive saliences, we computed the Pearson’s correlation coefficient between the original data and their projection onto the respective saliences, which results in the so-called brain and cognitive loadings (Kebets et al., 2019). A large positive (or negative) loading for a particular brain (cognitive) feature indicates a greater contribution of that feature to the multivariate correlation pattern. The statistical significance of the multivariate correlation patterns was assessed with permutation testing (1,000 permutations, correlation patterns with *p* < 0.05 after *FDR* correction were deemed significant). The reliability of brain and cognitive loadings for the significant correlation patterns was assessed with bootstrapping (500 random data resamplings) and computing standard scores with respect to the bootstrap distribution (loadings were considered reliable for absolute standard score > 3) (Krishnan et al., 2011; Zöller et al., 2019). For the PLSC analyses, missing cognitive scores were imputed using the 4-nearest-neighbor method.

All the analyses were performed with MATLAB (The MathWorks, Inc., version R2019b).

## Results

### Subjects and Cognitive Profiles

**FIGURE 1:**
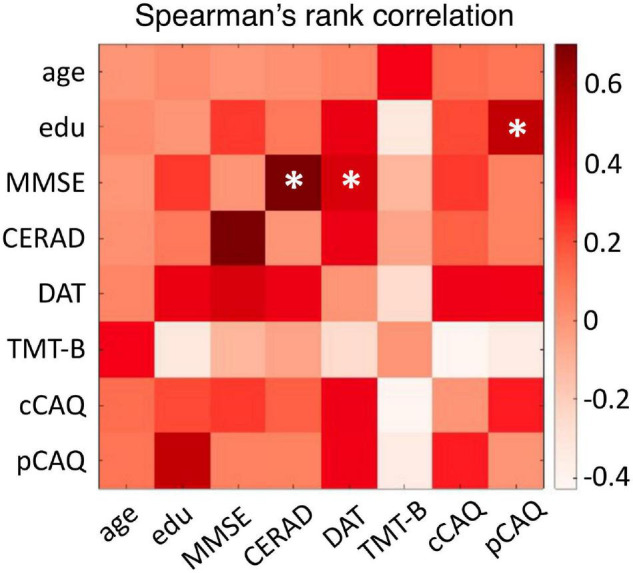
Relationships between cognitive and cognitive reserve scores. The matrix entries represent the Spearman’s rank correlation coefficient between cognitive performances, cognitive reserve, age and education level. Correlations surviving multiple comparison correction (*FDR* < 0.05) are indicated with an asterisk. Edu, education level; MMSE, Mini-Mental State Examination; CERAD, total score of the Consortium to Establish Registry for Alzheimer’s Disease; DAT, letters D-A-T fluency test; TMT-B, Trail Making Tests B score; cCAQ, current engagement in cognitively demanding activities; pCAQ, past engagement in cognitively demanding activities.

We investigated the spectral and functional connectivity profiles of MEG data recorded in 35 CN and 11 CI oldest-old subjects. The demographic, clinical and cognitive characteristics of the two groups, and the related statistical comparisons, are reported in Table 1. There was a significant difference between the two groups in terms of age [Student’s *t*-test, *t*(44) = 2.03, *p* = 0.048, CI < CN, difference of the means = 1.3 years] and gender [proportionally fewer women in the CI group, Chi-square test, C^2^(1, *N* = 46) = 4.82, *p* = 0.028], and no significant difference in years of education or GDS TOTAL score. By definition CI subjects had significantly lower MMSE total [*F*(1, 42) = 60.73, *p* < 10^–9^] and CERAD total [*F*(1, 42) = 19.15, *p* = 0.000073] scores, indicating overall cognitive impairment and reduced episodic memory performances compared to CNs, when taking into account the effects of age and gender. There were no differences between CNs and CIs with respect to letter fluency and TMT-B scores. At the time of this study, CI subjects engaged less frequently in cognitively demanding activity compared to CN subjects [cCAQ, *F*(1, 40) = 5.03, *p* = 0.030]. CN and CI oldest-old subjects did not differ in terms of cognitive reserve (i.e., there was no difference between CNs and CIs with respect to pCAQ scores). The rank correlations between age, years of education, cognition, and cognitive reserve scores in the whole groups of subjects are reported in Figure 1. There were statistically significant (*FDR* < 0.05) positive correlations between education level and pCAQ [(44) = 0.54, *p* = 0.0010]; verbal fluency and MMSE [(44) = 0.70, *p* < e^–7^); verbal fluency and cCAQ (ρ(44) = 0.45, *p* = 0.0016]; CERAD total and MMSE [ρ(44) = 0.40, *p* = 0.0062]. The pCAQ score was also positively correlated with the verbal fluency [(44) = 0.36, *p* = 0.020], but this association did not survive multiple comparison correction.

**TABLE 1:** Demographic and cognitive characteristics.

	CN (*n* = 35)	CI (*n* = 11)	*p*-values
**Demographic and clinical indicators**			
Age, years	92.2 (1.8)	90.9 (1.9)	0.048*
Gender, F/M	19/16	1/10	0.028*
Education, years	12.5 (4.7)	12.4 (4.2)	0.94
GDS TOTAL	1.7 (1.6)	3.0 (2.3)	0.057
**Cognition**			
MMSE, points	28.3 (1.1)	23.2 (3.4)	<e-9**
DAT fluency, number	28.2 (7.9)	23.0 (10.9)	0.090
TMT-B, seconds	268 (125)	217 (105)	0.15
CERAD TOTAL, words	16.6 (3.5)	11.4 (3.4)	0.000073**
**Cognitive engagement**			
cCAQ, points	3.2 (0.6)	2.6 (1.0)	0.03*
pCAQ, points	2.5 (0.6)	2.8 (0.6)	0.49

*Column 1: demographic, clinical and cognitive indicators. Columns 2 and 3: group-mean (standard deviation) values for continuous variables for the 35 cognitively normal (CN) and 11 cognitively impaired (CI) subjects. Column 4: p-values for statistical comparisons between CN and CI groups (one-way ANOVA for continuous and interval variables; chi-square test for categorical variables).*

*p < 0.05;

**p < 0.001. GDS TOTAL score was missing for 1 CI subject; TMT-B for 8 CN and 5 CI subjects; pCAQ for 1 CN and 1 CI subject; cCAQ for 1 CN and 1 CI subject. Reported statistics are based on available data. GDS, Geriatric Depression Scale; MMSE, Mini-Mental State Examination; CERAD, total score of the Consortium to Establish Registry for Alzheimer’s Disease; DAT, letters D-A-T fluency test; TMT-B, Trail Making Tests B score; cCAQ, current engagement in cognitively demanding activities; pCAQ, past engagement in cognitively demanding activities.

### Spectral Features in the Theta and Beta Bands Are Altered in Cognitively Impaired Oldest-Old Subjects

Spectral features of the CN and CI MEG were quantified with the IPF and the relative band power (RBP) in six frequency bands, both at the whole-brain and regional levels. Before computing IPF and RBP values, we verified that there was no significant difference in global power (i.e., average over all the 80 brain regions; [*F*(1, 42) = 0.06, *p* = 0.81] or total power estimated over the occipital regions only [*F*(1, 42) = 1.27, *p* = 0.27, Supplementary Table 1] between the CN and CI groups.

**FIGURE 2:**
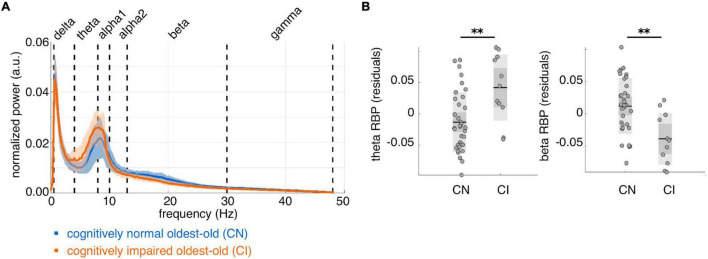
Whole-brain average spectral properties of cognitively normal and impaired oldest-old subjects. **(A)** Group-average power spectral density curves over 80 cortical regions of interest, for cognitively normal (*n* = 35, blue curve) and cognitively impaired (*n* = 11, orange curve) subjects. Solid lines represent the group means; shaded areas represent ± 1 standard deviation interval. **(B)** Distributions of whole-brain relative band power (RBP) in the theta and beta band, after correction for age and gender. ^**^p < 0.001 for CI-CN comparison.

On average, CI subjects had lower IPF than CN subjects, but this difference did not reach statistical significance (mean ± std IPF: CN = 9.1 ± 0.8 Hz, CI = 8.7 ± 0.3 Hz; [*F*(1, 42) = 2.44, *p* = 0.13]. We found significantly higher whole-brain theta RBP [*F*(1, 42) = 14.54, *p* = 0.00044, *d* = 1.15] and lower beta RBP [*F*(1, 42) = 16.82, *p* = 0.00018, *d* = –1.23] in CI compared to CN subjects (Figure 2). Moreover, the individual theta and beta RBP values were strongly negatively correlated across subjects [linear correlation coefficient *r*(44) = –0.79, *p* < e-10], suggesting an overall shift of the average MEG spectrum toward the lower frequencies in CI subjects. This effect is qualitatively illustrated by the group-average power spectral density curves in Figure 2A. There was also a significant decrease of whole-brain gamma RBP in CI compared to CN subjects, but this effect had smaller effect size than was the case for the theta and beta bands [*F*(1, 42) = 4.19, *p* = 0.047, *d* = –0.63]. No significant CI-CN whole-brain RBP differences were found in the delta, alpha1 or alpha2 frequency band.

**FIGURE 3:**
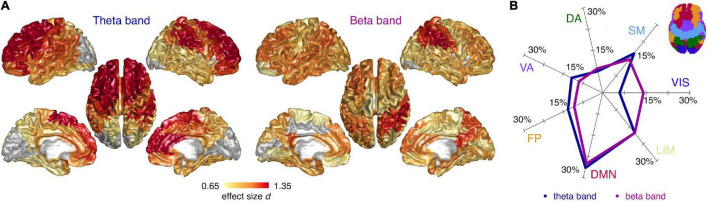
Cortical distribution of spectral differences between cognitively normal and impaired subjects. **(A)** Cortical surface plots of local effect size (Cohen’s *d*) for statistically significant (*FDR* < 0.05) CI-CN comparisons of source relative band power (RBP), in the theta and beta frequency band (ANCOVA analyses including age and gender covariates). Cortical regions not surviving multiple comparison correction are represented in gray. **(B)** Percentage effect size contributions in the 7 RSNs, for the theta and beta RBP. The percentage effect size contribution for each RSN was assessed by normalizing the effect size sum over the regions belonging to each RSNs by the sum of all regions’ effect size, and considering only the regions significantly different between cognitively normal and impaired subjects. Inset: schematic representation of the 7 RSNs on the cortical surface. VIS, visual; SM, sensorimotor; DA, dorsal attention; VA, ventral attention; FP, fronto-parietal; DMN, default mode; LIM, limbic network.

Next, we investigated the spectral properties of CN and CI time-series at the level of the individual cortical regions. We found spatially diffuse CI-CN RBP alterations in the theta and beta band with 73 and 77 regions surviving multiple comparison correction, respectively (*FDR* < 0.05). In the theta band, RBP was higher in CI compared to CN subjects in the frontal lobe, including superior frontal and anterior cingulate cortices, in the primary and association somatosensory cortices, and, to a lesser extent, in the parietal and temporal lobes (no region showed lower theta RBP) (Figure 3A). In the beta band, RBP was lower in CI compared to CN subjects in superior parietal regions (including the postcentral gyrus), posterior cingulate/precuneus, dorsolateral prefrontal, and anterior cingulate cortices (no region showed higher beta RBP) (Figure 3A). Globally, these spectral alterations mainly involved the default mode network and, to a lesser extent, the limbic, somatomotor, and fronto-parietal resting state networks, in both the theta and beta band (Figure 3B). The visual cortex was spared in the theta bands but partially affected in the beta band.

### Functional Connectivity Is Similar Between Cognitively Normal and Impaired Subjects

We investigated possible CI-CN group-differences of functional connectivity values at whole-brain and cortical region level. At the whole-brain level, the average functional connectivity in the alpha2 band was decreased in CI compared to CN, with small effect size [*F*(1, 42) = 4.28, *p* = 0.045, *d* = –0.64; *F*(1, 42) = 2.36, *p* = 0.13, *d* = –0.47 when also covarying for the alpha2 RBP]. There was no CI-CN difference of average functional connectivity in the other frequency bands. Similarly, no CI-CN comparison of functional connectivity at the level of single brain regions survived multiple comparison correction (*FDR* < 0.05) in any frequency band.

### Magnetoencephalography Brain Features Relate to Cognition and Cognitive Reserve in Oldest-Old Subjects

The cognitive profile of individual subjects was characterized in terms of overall cognitive ability (MMSE score), executive control (letter fluency), processing speed (TMT-B score) and episodic memory (CERAD total score). Moreover, we considered the lifelong engagement in cognitively demanding activity as possible protective factors for cognitive impairment and proxy for subjects’ cognitive reserve. We investigated multivariate linear relationships between whole-brain spectral or functional connectivity features, and cognition, cognitive reserve, education level and age with two PLSC analyses. The analyses were performed on the whole group of 46 subjects (i.e., considering both CI and CN subjects) and replicated in the CN group (we did not repeat the analyses in the CI group given the small sample size).

**FIGURE 4:**
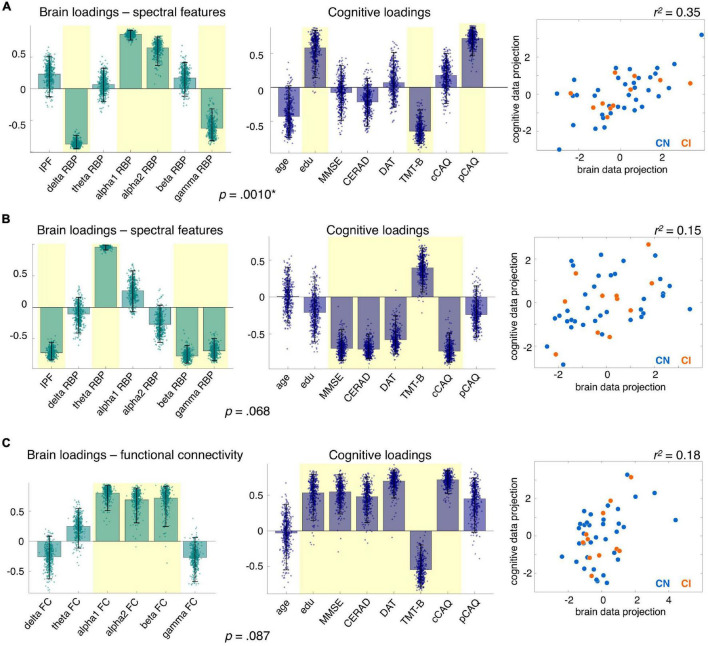
Multivariate correlation patterns between electrophysiological and cognitive features. Each panel represents, from left to right: (i) the brain loadings, (ii) the cognitive loadings, and (iii) the data projection onto the brain and cognitive saliences for the first **(A)** and second **(B)** significant multivariate correlation patterns between spectral and cognitive features, and for the multivariate pattern between functional connectivity and cognitive features **(C)** from partial least square correlation (PLSC) analyses. In the loading plots, bars and dots represent the average and dispersion of brain and cognitive loadings over 500 bootstraps; loadings reliably different from zero are shaded in yellow. *P*-values for the multivariate correlation patterns are reported below the loading bar plots (* indicates pattern surviving multiple comparison correction at *FDR* < 0.05). In the scatter plots on the right, each dot represents brain and cognitive data of a single subject projected onto the corresponding PLSC saliences, with cognitively normal (CN) and impaired (CI) subjects represented in light blue and orange, respectively. The r-squared between the brain and cognitive data projection onto the PLSC saliences is reported above each scatter plot and quantifies the amount of cognitive scores’ variance explained by the spectral or functional connectivity features.

Concerning the spectral features, the PLSC analysis extracted by construction 7 multivariate correlation patterns, one of which was statistically significant (*p* = 0.0010; *FDR* < 0.05). On an exploratory basis, we also report a second multivariate correlation pattern with *p* = 0.68. The brain and cognitive loadings associated with the two patterns are shown in Figures 4A,B, with loadings that were reliably different from zero highlighted in yellow. The first multivariate pattern shows an association between higher cognitive reserve (larger pCAQ score and education level, while taking into account the age) and processing speed, and a spectral signature characterized by less power in the delta and gamma band and more power in the alpha band (Figure 4A). The second multivariate pattern mirrors the CI-CN differences reported above (Figure 2) and suggests an association between poorer cognitive performances (including lesser current involvement in cognitively demanding activities, i.e., lower cCAQ) and slowing down of brain oscillations, particularly involving the beta and theta band (Figure 4B).

Concerning the functional connectivity features, none of the multivariate correlation patterns survived multiple comparison correction. However, we report on an exploratory basis the correlation pattern with the smallest *p*-value (*p* = 0.087), which suggests a possible relationship between better cognitive performance and stronger functional connectivity in the alpha and beta band (Figure 4C). All PLSC results were consistent when analyses were performed on CN participants only (Supplementary Figure 5), suggesting that the brain-cognition associations reflect a continuum over cognitive decline stages and are not driven by just the cognitively impaired individuals.

## Discussion

This study represents the first characterization of neuronal oscillations’ spectral features and amplitude coupling with respect to cognition and lifelong engagement in cognitive activity in oldest-old participants using MEG. Compared to cognitively normal subjects, those with cognitive impairments showed extended alterations of relative power in the theta and beta band, indicating a global slowing of cortical oscillations. The source-level power alterations heavily involved the frontal lobe in the theta band and extended to fronto-parietal and visual areas in the beta band, with an overall predominant involvement of the default mode network. Spectral and, to a lesser extent, functional connectivity features related to cognitive traits. In the spectral domain, two multivariate correlation patterns were discussed, one mirroring the spectral changes observed in cognitively impaired participants with lower (higher) power content in the theta (beta) band associated with better cognitive performances (trend-level, *p* = 0.068). The main multivariate correlation pattern (*p* = 0.0010) revealed an association between spectral content in the delta, alpha, and gamma band, and cognitive reserve approximated with the lifelong (past) engagement in cognitively demanding activity. Finally, better cognitive performances were marginally associated with overall stronger functional connectivity in the alpha and beta band.

Our finding of higher theta and lower beta power in cognitively impaired oldest-old subjects suggests that the association between electrophysiological changes and cognitive impairment is substantially similar in oldest-old participants and individuals younger than 85 years.

Younger old-adults with prodromal AD, early onset AD or typical-onset AD show widespread power increases of electrophysiological signals in lower frequency bands (delta and theta band) and power decreases in higher frequency bands (alpha and beta band) compared to normal aging adults, indicating a global slowing of resting-state activity (Dauwels et al., 2010; Micanovic and Pal, 2014; Engels et al., 2016, 2017; Gouw et al., 2017; Babiloni et al., 2020a). This finding is highly consistent in literature, and it is here extended to cognitively impaired oldest-old with probable late-onset AD or aMCI. It should be noted, however, that the slowing of cortical oscillations is observed not only in AD, but also in multiple pre-dementia and dementia forms (notably, dementia with Levy Bodies) (Dauwan et al., 2016; van der Zande et al., 2020), as well as in normal aging (Knyazeva et al., 2018). In our sample, there was a small but significant age difference between cognitively normal and cognitively impaired subjects, but the latter were on average younger than the former. It is therefore unlikely that the slowing down of cortical rhythms observed in our cognitively impaired sample was due to physiological aging rather than neurodegenerative processes. In further support of this interpretation, lower IPF, larger relative power in the theta band and lower power in higher frequency bands (beta, gamma) were weakly associated with worse overall cognitive performances, memory, executive control, processing speed, and current engagement in cognitively demanding activity both in the whole sample and in the cognitively normal group only, suggesting a relationship between cortical slowing and cognition that is independent from clinical classification. This is consistent with previous findings that have linked increased theta power with decreased cognitive functioning in healthy older adults (Mitchell et al., 2008; Stomrud et al., 2010; Finnigan and Robertson, 2011). Moreover, baseline theta power predicts longitudinal cognitive decline and conversion to dementia in younger old-adults (Prichep et al., 2006; Gouw et al., 2017; Rossini et al., 2020). Finally, previous works have associated theta (but also delta and alpha) power with clinical symptoms and global cognitive status in AD patients (Engels et al., 2016; Gouw et al., 2017). In our study, it was not possible to investigate associations between electrophysiological and cognitive features specifically in the AD/aMCI group given the small sample size (only 11 out of 46 subjects were cognitively impaired). Therefore, it remains to be further investigated in a larger cohort whether the relationships between spectral features and cognition in this age range are diagnosis-dependent (Vlahou et al., 2014; Benwell et al., 2020) or reflect more generic neurodegenerative processes that lead to cognitive decline.

The cortical distributions of the theta and beta changes in cognitively impaired oldest-old participants largely overlapped in the frontal lobe with involvement of the default mode network, but showed distinct spatial patterns in posterior cortices.

Theta alterations were widespread and mainly involved the frontal lobe, while beta alterations extended to more posterior areas, including the visual cortices and showing relatively large effects in the precuneus and posterior cingulate regions. The superior parietal cortex was affected in both bands, in agreement with MEG findings in younger AD patients (Berendse et al., 2000; Engels et al., 2016, 2017). Globally, the power changes involved the default mode network, a brain system that includes medial (medial prefrontal and precuneus/posterior cingulate cortices), hippocampal and parietal regions (Raichle et al., 2001; Greicius et al., 2003; Andrews-Hanna et al., 2014; Raichle, 2015). Functional connectivity in the default mode network predicts cognitive abilities in healthy adults (Van Den Heuvel et al., 2009) and is strongly implicated in the pathophysiology of AD (Agosta et al., 2012). In AD and preclinical AD, default mode regions show early accumulation of amyloid-β and early neurodegeneration (Palmqvist et al., 2017; Sepulcre et al., 2017), possibly driven by high baseline activity levels (Buckner et al., 2009). Default mode regions in AD also show decreased synchronization of hemodynamic signals (weakened functional connectivity) as assessed with resting-state functional magnetic resonance imaging (rfMRI) (Myers et al., 2014; Pasquini et al., 2017). Interestingly, simultaneous EEG-rfMRI studies in healthy subjects specifically associate the amplitude of neuronal oscillations in the theta and beta frequency band to default mode network hemodynamic activity (Laufs et al., 2003; Scheeringa et al., 2008; Hlinka et al., 2010). Alterations of default mode hemodynamic activity and widespread changes of theta and beta rhythms could therefore be the manifestations of the same pathophysiological mechanisms, such as activity-dependent neurodegeneration (Buckner et al., 2009; Griffa and van den Heuvel, 2018; de Lange et al., 2019). Moreover, computational models demonstrate that activity-dependent degeneration of default mode regions can reproduce AD-like changes such as oscillatory slowing and loss of spectral power (de Haan et al., 2012). Yet, the subdivision of cortical regions into RSNs that we used in this work was derived from fMRI data (Yeo et al., 2011). It is not yet clear whether MEG functional activity shows the same RSNs (de Pasquale et al., 2010), especially in this age group, which deserves further investigation.

Previous studies on AD patients also report slower rhythms in the occipital lobe and visual areas in the alpha band (Engels et al., 2017; Babiloni et al., 2020a), which was not the case for our cohort. However, the alpha band was involved in terms of functional connectivity, with cognitively impaired oldest-old participants having lower alpha2 amplitude coupling at the whole-brain network level. This CI-CN difference partially related to the power content in the two groups, since covarying by the alpha2 band power decreased the effect size. Nonetheless, the functional-connectivity group-effect should not be disregarded because of the power contribution. Signal power is necessary to get functional connectivity, especially when connectivity is based on amplitude coupling, and the relationship between the two dimensions is non-trivial and may reflect underlying mechanisms (Tewarie et al., 2019). In addition, the PLSC analysis suggested a relationship between stronger functional connectivity in the alpha and beta band, and preserved cognitive performances, particularly in the executive domain. These results in oldest-old participants are in line with MEG literature showing decreased functional connectivity in AD (Berendse et al., 2000; Stam et al., 2002, 2006, 2009; Yu et al., 2017), but they remain preliminary considering the limited power of the study in relation to the small effects detected in the functional connectivity domain. Indeed, it should be noted that the effect sizes of the CI-CN group-differences and the linear associations with cognitive traits were more prominent in the spectral domain, highlighting the relevance of relatively simple electrophysiological measures in a clinical setting. Functional connectivity analyses on larger cohorts may nonetheless contribute to the understanding of neural mechanisms associated with specific cognitive dysfunctions -such as impairments in executive functioning- that strongly rely on network-level integration processes.

Participants underwent an interview reporting how often they engaged in common cognitively demanding activities that depend minimally on socioeconomic status (Landau et al., 2012). Lifestyle factors are considered as a proxy for cognitive reserve, defined as the adaptability of functional brain processes to cope with aging, brain insults or pathological processes (Stern, 2009; Stern et al., 2018). In particular, frequency of past and present engagement in cognitively demanding activities has been associated with lower amyloid-β accumulation in brain tissues (Landau et al., 2012), less hippocampal atrophy (Valenzuela et al., 2008), and lower dementia incidence (Valenzuela and Sachdev, 2006; Xu et al., 2019) in healthy older adults. However, in subjects aged 85 years or older, education, occupational complexity and engagement in cognitive and leisure activities do not predict cognitive decline nor the risk of 5-year incident dementia (Lavrencic et al., 2018; Hakiki et al., 2020), suggesting that cognitive reserve mechanisms may be age-dependent and become less effective in counterbalancing neurodegenerative processes (Nelson et al., 2019). In line with these observations, past engagement in cognitively demanding activities (pCAQ score) did not differ between cognitively normal and impaired oldest-old participants in our study, although higher pCAQ scores were weakly associated with better executive performances at the whole-group level. As expected, higher pCAQ (but not cCAQ) scores were also associated with higher educational level, a component of the cognitive reserve construct (Nucci et al., 2012). On the contrary, cognitively impaired participants tended to engage less frequently in cognitive activities than cognitively normal ones at the time of the study. The frequency of current engagement in cognitively demanding activities may therefore better reflect the present cognitive status rather than cumulate cognitive reserve. Our and literature findings converge on the hypothesis that education and sociobehavioral lifestyle habits including lifelong (past) engagement in cognitively demanding activities may serve as protective factors for cognitive decline in younger-old, but that this beneficial effect may be progressively less prominent in oldest-old subjects who, possibly, face distinct or more severe pathophysiological mechanisms.

In support of this hypothesis, we found that cognitive reserve in oldest-old participants was associated with a specific spectral signature involving delta, alpha and gamma band, in contrast to the spectral changes associated with cognitive performances, which mainly involved the theta and beta band. In particular, higher cognitive reserve (higher pCAQ scores and education level while accounting for age) related to lower (higher) cortical oscillation power in the delta (alpha) band, combined with lower power in the gamma band. This finding nicely corroborates a recent sensor-level EEG study that identified higher alpha amplitudes in (amyloid negative) older adults (mean age 75 years) with subjective cognitive complaints and higher educational level compared to those with lower education level (Babiloni et al., 2020b). It is well known that posterior resting-state alpha is progressively reduced with aging, which may partially be linked to a deterioration of the cholinergic system (Wan et al., 2019). Our results suggest that lifestyle factors may compensate this process, even at advanced age, resulting in stronger alpha activity at rest. However, we have also shown that the relationship between cognitive reserve and spectral features is independent from memory and executive control performance, which was taken into account through the multivariate nature of our analyses and replication of results in the cognitively normal group. Yet, the spectral signature of cognitive reserve might change as a function of the pathological substrate underlying cognitive decline (Babiloni et al., 2021). Considering the small size of the CI group, it was not possible to perform reliable correlation analyses within this group. Further research is needed to elucidate the interplay between the distinct electrophysiological mechanisms reflecting cognitive reserve, cognitive decline, and pathological load, particularly in an age segment - the oldest-old- for which dementia risk and protective factors identified in younger subjects may not be valid. Taken together, our results indicate that functional adaptability mechanisms associated with cognitive reserve (lifelong engagement in cognitive activity) are present in the oldest-old and expressed in specific electrophysiological signatures, but that they are less effective in limiting cognitive decline.

This study has some limitations that should be noted. First, the sample size is relatively small and absence of statistically significant findings might relate to limited statistical power. However, one should consider that the recruitment of oldest-old subjects in research programs is challenging and few neuroimaging data are available for oldest-old participants (Legdeur et al., 2018). Second, in this study measures of brain pathology, such as biomarkers for amyloid, tau, and cerebrovascular pathologies, were not taken into account. Third, the subjects’ cognitive profile was condensed in few cognitive scores probing executive control, processing speed and episodic memory, mainly to accommodate the limited statistical power linked to the small sample size. However, further analyses are needed to fully explore the relationship between cognitive dimensions and MEG features. For example, executive control is a complex construct that is only partially captured by the phonemic verbal fluency (DAT scores) (Jurado and Rosselli, 2007; Friedman and Miyake, 2017; Vallesi, 2021). Finally, individual levels of cognitive reserve were approximated with a self-reported questionnaire on past engagement in cognitively demanding activities. Self-reporting may be poorly reliable, particularly in the oldest-old age range, and additional sociobehavioral proxies of cognitive reserve could be used in future studies (Stern et al., 2018).

To conclude, in this work we have shown that cognitive impairments in oldest-old subjects are associated with a slowing of theta/beta oscillatory brain activity converging onto the default mode network. In the same subjects, a distinct spectral signature involving the delta, alpha and gamma band is associated with cognitive reserve mechanisms, which, however, may be ineffective in preserving cognitive performances in this age range. Future studies should further investigate how these brain functional changes relate to underlying neuropathological factors and to functional adaptive mechanisms that are possibly specific to this age range.

## Data Availability Statement

The datasets presented in this article are not readily available because they are already part of the EMIF-AD 90 + study. Requests to access the datasets should be directed to AH.

## Ethics Statement

The studies involving human participants were reviewed and approved by the local Medical Ethics Review Committee of the Amsterdam UMC. The patients/participants provided their written informed consent to participate in this study.

## Author Contributions

AG, AH, CS, and PV co-designed the study. AG performed the analyses and wrote the first version of the manuscript. NL and MB took care of data curation and EMIF-AD 90 + database. AH, CS, PV, MH, AG, and NL were involved in the interpretation and discussion of results. All authors contributed to manuscript revision, read, and approved the submitted version.

## Conflict of Interest

The authors declare that the research was conducted in the absence of any commercial or financial relationships that could be construed as a potential conflict of interest.

## Publisher’s Note

All claims expressed in this article are solely those of the authors and do not necessarily represent those of their affiliated organizations, or those of the publisher, the editors and the reviewers. Any product that may be evaluated in this article, or claim that may be made by its manufacturer, is not guaranteed or endorsed by the publisher.
