# Supplementary material for: Magnetoencephalography Brain Signatures Relate to Cognition and Cognitive Reserve in the Oldest-Old: The EMIF-AD 90 + Study
Source: Front Aging Neurosci. 2021 Nov 25;13:746373. doi: 10.3389/fnagi.2021.746373 (PMC8656941; doi:10.3389/fnagi.2021.746373)
Supplement: Supplementary file 1 [file Data_Sheet_1.docx]

**SUPPLEMENTARY MATERIAL**

**MEG spectral and functional connectivity signatures relate to cognition and**

**cognitive reserve in the oldest-old: The EMIF-AD 90+ study**

Alessandra Griffa^a,b,c^, Nienke Legdeur^d^, Maryam Badissi^d^, Martijn P. van den Heuvel^e^, Cornelis J. Stam^c^, Pieter Jelle Visser^d,f^, Arjan Hillebrand^c^

a. Department of Clinical Neurosciences, Division of Neurology, Geneva University Hospitals and Faculty of Medicine, University of Geneva, Geneva, Switzerland

b. Institute of Bioengineering, Center of Neuroprosthetics, École Polytechnique Fédérale De Lausanne (EPFL), Geneva, Switzerland

c. Amsterdam UMC, Vrije Universiteit Amsterdam, Department of Clinical Neurophysiology and MEG Center, Amsterdam Neuroscience, De Boelelaan 1117, Amsterdam, The Netherlands

d. Alzheimer Center Amsterdam, Department of Neurology, Amsterdam Neuroscience, Vrije Universiteit Amsterdam, Amsterdam UMC, Amsterdam, The Netherlands

e. Dutch Connectome Lab, Department of Complex Trait Genetics, Center for Neuroscience and Cognitive Research, Amsterdam Neuroscience, Vrije Universiteit Amsterdam, Amsterdam UMC, Amsterdam, The Netherlands

f. Department of Psychiatry & Neuropsychology, School for Mental Health and Neuroscience, Maastricht University, Maastricht, The Netherlands

**Contents**

**SI.1.** Automated adjustment of the origin for temporal signal space separation (tSSS)

**SI.2.**  Epoch selection procedure

**Table S1.** Regions of interest

**Figure S1.** Automatic adjustment of source coordinates for tSSS

**Figure S2.** No relationship between head position and spectral features

**Figure S3.** Epoch selection

**Figure S4.** Group-average functional connectivity matrices

**Figure S5.** Multivariate correlation patterns between electrophysiological and cognitive features evaluated in 35

cognitively normal oldest-old subjects

**References** References of SI section

**SI.1. Automated adjustment of the origin for temporal signal space separation (tSSS)**

Sensor-level MEG signals are often corrupted by artefacts. These contributions, including external magnetic interferences (e.g., elevators, traffic) and physiological artefacts (e.g., cardiac activity, muscular activity), can be attenuated using temporal signal-space separation (tSSS) (Hari and Puce, 2017). SSS models the magnetic field $\bar{b}$ perceived at the sensor level as the sum of three components: (i) the brain signals $\bar{b_{in}}$ originating inside a sphere of interest (which ideally corresponds to the sensor-array sphere and is concentric to the head sphere, Figure S1A), (ii) the external artefacts $\bar{b_{out}}$ originating outside the sphere of interest, and (iii) the artefacts $\bar{n}$ originating from sources of interference located very close to the sensors (Taulu et al., 2004, 2005; Taulu and Simola, 2006):

$$\bar{b}=\bar{b_{in}}+\bar{b_{out}}+ \bar{n}$$

The sensors’ geometry and Maxwell’s equations are used to estimate the three components $\bar{b_{in}}$, $\bar{b_{out}}$, $\bar{n}$ by expressing the sensor signals $\bar{b}$ as harmonic series, while imposing the temporal independence of $\bar{b_{in}}$ and $\bar{b_{out}}$ (‘temporal’ extension of the signal space separation approach, allowing to disentangle the interferences originating close to the sensors, or large artefacts that contribute to both $\bar{b_{in}}$ and $\bar{b_{out}}$) (Taulu and Hari, 2009). A key element for an optimal SSS-based artefact attenuation is the definition of the sphere of interest *S_in_*, which should perfectly contain the brain volume and approximatively coincide with the sensor-array sphere (Figure S1A). The center of the sphere of interest is then set as the origin of the spherical harmonics’ expansion in the SSS formulation.

Practically, in this work we used the tSSS implementation in Elekta’s *MaxFilter* software (Elekta Neuromag Oy, Helsinki, Finland). The researcher is required to set a few tSSS parameters, including the center of the sphere of interest (origin of the spherical harmonics expansion). The radius of the sphere of interest is then automatically set equal to the average distance between the defined sphere center and the MEG sensors.

In an ideal case, the head of the subject is perfectly positioned inside the MEG helmet: The center of the sphere of interest *S_in_* coincides with the center of the sensor-array sphere *S_sensors_* and is concentric to the head sphere *S_head_* (‘Optimal head position’, Figure S1B). *S_in_* perfectly contains the brain volume and excludes areas outside the head, resulting in an optimal tSSS artefact correction. In a less ideal case, the head of the subject is not perfectly positioned inside the MEG helmet and *S_head_* is not completely contained within *S_sensors_* (‘Suboptimal head position’, Figure S1B). This is a common situation that can occur because of subject movement, poor initial positioning of the subject inside the MEG helmet, or inability to fit the head completely inside the helmet (e.g., due to a large head). In this case, setting the center of *S_in_* (origin of the spherical harmonics’ expansion) equal to the center of *S_sensors_* would result in the exclusion of part of the brain volume and in the possible tSSS-based suppression of neuronal components. On the other hand, setting the center of *S_in_* equal to the center of *S_head_* would result in a large sphere of interest (with radius equal to the average distance between the *S_head_* center and the MEG sensors) including a significant portion of space external to the subject head (Figure S1B). This setting can lead to a possible wrong attribution of artefactual signals to the $\bar{b_{in}}$ component, resulting in poor tSSS outcome (i.e. noisy data).

To achieve a trade-off between these two scenarios (suppression of neuronal components or inclusion of artefactual components) and optimize the tSSS-based artefact suppression, we set the center of *S_in_* equal to the midpoint of the segment linking the centers of *S_sensors_* and *S_head_* (‘Automatic selection of *S_in_* center’, Figure S1B). The center of *S_in_* was therefore automatically tuned, independently for each subject included in this study. This approach results in reasonably good tSSS results, as assessed by visual inspection of MEG data before and after the tSSS processing step.

Moreover, to conceptually validate our approach, we investigated the variation of the signals’ average power content after MEG data preprocessing as a function of the *S_in_* choice. Data were preprocessed using different *S_in_* center coordinates, starting from the *S_head_* center coordinates and moving toward the sensors’ array; band-passed filtered to the frequency band 0.5-48 Hz; source-reconstructed (i.e., projected to 80 cortical regions of interest); and Fourier-transformed to obtain the average power spectral density across the cortical sources. We analyzed the average power content of the reconstructed signals, defined as the integral of the average power spectral density across all brain sources.

We observed that in all cases the average power content decreases when moving the *S_in_* center toward the sensors’ array. Indeed, when the *S_in_* center becomes very close to the sensors’ array, signals of neuronal origin are suppressed. However, the exact shape of the average power content curve as a function of the *S_in_* center coordinates depends on the subject’s head position with respect to the MEG sensors (Figure S1C, S1D). For subjects with a good head positioning inside the MEG helmet, the average power content remains stable when moving *S_in_* around the *S_head_* and *S_sensors_* center; it then starts to drop when moving *S_in_* closer to the sensors’ array (grey area in Figure S1C). For subjects with suboptimal head positioning inside the MEG helmet, the average power curve tends to show the highest values in an area between the *S_head_* and the *S_sensors_* center, arguably when *S_in_* includes the largest portion of the true neuronal signal while minimizing external artefacts (an example is given in Figure S1D). We observe that it is unlikely that the larger power values obtained when moving *S_in_* between *S_head_* and *S_center_* are due to a significant inclusion of external artefacts, since the power spectra do not show suspicious low-frequency or high-frequency contributions (Figure S1D). Setting the *S_in_* center between the *S_sensors_* and the *S_head_* (‘Automatic selection of *S_in_* center’) delivers in all cases high average power content of the source-signal (orange bars in Figure S1C, S1D), suggesting that our approach achieves a good trade-off between neuronal signal inclusion and artifactual signal suppression when the subject’s head position in the MEG helmet is suboptimal (as well as when the subject is optimally placed).

For the analyses reported in the main text, MEG data were tSSS-processed using the automatic selection of the center of the sphere of interest, and subsequently projected to source space (80 cortical regions of interest). In this case, we did not find any statistically significant relationship between the subjects’ head position (quantified as the distance between the center of the head sphere and the center of the sensor array sphere) and spectral measures (peak frequency, the relative alpha1-power over the occipital channels, and global power) (Figure S2).

**SI.2. Epoch selection procedure**

Source-level, preprocessed MEG data were subdivided into epochs of 13.1 seconds (16384 samples). An automatic epoch selection procedure was developed to discard (1) epochs with extreme values in the temporal domain and therefore likely corrupted by artefacts such as (eye) movements or swallowing; and (2) epochs during which the subject was likely to be drowsy.

Artefactual epochs with extreme values in the temporal domain were identified by computing the skewness of the time series amplitude distribution at the level of the single brain sources. Epochs were discarded when the average skewness over the ten sources (i.e., 12% of sources) with largest skewness exceeded the threshold value of 10. Note that a skewness of 0 indicates a perfectly symmetric distribution with no extreme values in the temporal domain; the skewness threshold was empirically set equal to 10.

Epochs during which the subject was likely to be drowsy were identified by considering the individual peak frequency (spectral peak in the range 4-13Hz) and the relative power content in the alpha1 band (8-10 Hz) over the occipital channels. It is well known that as a subject drowses, the posterior alpha activity is first more prominent than during the awake stage, and as the subject moves toward falling asleep, it starts to become disorganized (Hari and Puce, 2017). These effects are reflected in individual peak frequency outliers and drops in occipital alpha power content (Figure S3A, S3B). Epochs with a peak frequency value outside the range mean ± 1.282 standard deviations (mean and standard deviation computed over all epochs of a single subject) were therefore discarded. The range mean ± 1.282 standard deviations corresponds approximatively to the 90% of the data (i.e., of the epochs). Afterwards, in order to avoid possible drowsiness biases and select an equivalent amount of data across subjects, the 8 with highest individual peak frequency and occipital alpha1 power content were selected for each subject (Figure S3C).

**Table S1. Regions of interest**

Column 1: numerical ID of the cortical regions of interest. Column 2: anatomical labels of the cortical regions of interest, according to the AAL atlas (Tzourio-Mazoyer et al., 2002; Gong et al., 2009; Yeo et al., 2011); ‘_L’ indicates left hemisphere, ‘_R’ indicates right hemisphere. Column 3: assignment of each cortical regions to one of the 7 resting state networks defined in (Yeo et al., 2011) - visual (14 ROIs), sensorimotor (12 ROIs), dorsal attention (6 ROIs), ventral attention (8 ROIs), limbic (14 ROIs), fronto-parietal (8 ROIs), and default mode network (18 ROIs)-. Column 4: cortical regions belonging to the occipital lobe.

| **Numerical IDs** | **Anatomical labels** | **Resting state network** | **Occipital region?** |
| --- | --- | --- | --- |
| 1 | Rectus_L | Limbic |  |
| 2 | Olfactory_L | Limbic |  |
| 3 | Frontal_Sup_Orb_L | Limbic |  |
| 4 | Frontal_Med_Orb_L | Default mode |  |
| 5 | Frontal_Mid_Orb_L | Fronto-parietal |  |
| 6 | Frontal_Inf_Orb_L | Default mode |  |
| 7 | Frontal_Sup_L | Default mode |  |
| 8 | Frontal_Mid_L | Fronto-parietal |  |
| 9 | Frontal_Inf_Oper_L | Dorsal Attention |  |
| 10 | Frontal_Inf_Tri_L | Fronto-parietal |  |
| 11 | Frontal_Sup_Medial_L | Default mode |  |
| 12 | Supp_Motor_Area_L | Ventral Attention |  |
| 13 | Paracentral_Lobule_L | Sensorimotor |  |
| 14 | Precentral_L | Sensorimotor |  |
| 15 | Rolandic_Oper_L | Sensorimotor |  |
| 16 | Postcentral_L | Sensorimotor |  |
| 17 | Parietal_Sup_L | Dorsal Attention |  |
| 18 | Parietal_Inf_L | Fronto-parietal |  |
| 19 | SupraMarginal_L | Ventral Attention |  |
| 20 | Angular_L | Default mode |  |
| 21 | Precuneus_L | Default mode |  |
| 22 | Occipital_Sup_L | Visual | x |
| 23 | Occipital_Mid_L | Visual | x |
| 24 | Occipital_Inf_L | Visual | x |
| 25 | Calcarine_L | Visual | x |
| 26 | Cuneus_L | Visual | x |
| 27 | Lingual_L | Visual | x |
| 28 | Fusiform_L | Visual |  |
| 29 | Heschl_L | Sensorimotor |  |
| 30 | Temporal_Sup_L | Sensorimotor |  |
| 31 | Temporal_Mid_L | Default mode |  |
| 32 | Temporal_Inf_L | Dorsal Attention |  |
| 33 | Temporal_Pole_Sup_L | Limbic |  |
| 34 | Temporal_Pole_Mid_L | Limbic |  |
| 35 | ParaHippocampal_L | Limbic |  |
| 36 | Cingulum_Ant_L | Default mode |  |
| 37 | Cingulum_Mid_L | Ventral Attention |  |
| 38 | Cingulum_Post_L | Default mode |  |
| 39 | Insula_L | Ventral Attention |  |
| 40 | Rectus_R | Limbic |  |
| 41 | Olfactory_R | Limbic |  |
| 42 | Frontal_Sup_Orb_R | Limbic |  |
| 43 | Frontal_Med_Orb_R | Default mode |  |
| 44 | Frontal_Mid_Orb_R | Fronto-parietal |  |
| 45 | Frontal_Inf_Orb_R | Default mode |  |
| 46 | Frontal_Sup_R | Default mode |  |
| 47 | Frontal_Mid_R | Fronto-parietal |  |
| 48 | Frontal_Inf_Oper_R | Dorsal Attention |  |
| 49 | Frontal_Inf_Tri_R | Fronto-parietal |  |
| 50 | Frontal_Sup_Medial_R | Default mode |  |
| 51 | Supp_Motor_Area_R | Ventral Attention |  |
| 52 | Paracentral_Lobule_R | Sensorimotor |  |
| 53 | Precentral_R | Sensorimotor |  |
| 54 | Rolandic_Oper_R | Sensorimotor |  |
| 55 | Postcentral_R | Sensorimotor |  |
| 56 | Parietal_Sup_R | Dorsal Attention |  |
| 57 | Parietal_Inf_R | Fronto-parietal |  |
| 58 | SupraMarginal_R | Ventral Attention |  |
| 59 | Angular_R | Default mode |  |
| 60 | Precuneus_R | Default mode |  |
| 61 | Occipital_Sup_R | Visual | x |
| 62 | Occipital_Mid_R | Visual | x |
| 63 | Occipital_Inf_R | Visual | x |
| 64 | Calcarine_R | Visual | x |
| 65 | Cuneus_R | Visual | x |
| 66 | Lingual_R | Visual | x |
| 67 | Fusiform_R | Visual |  |
| 68 | Heschl_R | Sensorimotor |  |
| 69 | Temporal_Sup_R | Sensorimotor |  |
| 70 | Temporal_Mid_R | Default mode |  |
| 71 | Temporal_Inf_R | Dorsal Attention |  |
| 72 | Temporal_Pole_Sup_R | Limbic |  |
| 73 | Temporal_Pole_Mid_R | Limbic |  |
| 74 | ParaHippocampal_R | Limbic |  |
| 75 | Cingulum_Ant_R | Default mode |  |
| 76 | Cingulum_Mid_R | Ventral Attention |  |
| 77 | Cingulum_Post_R | Default mode |  |
| 78 | Insula_R | Ventral Attention |  |
| 79 | Hippocampus_L | Limbic |  |
| 80 | Hippocampus_R | Limbic |  |

**Figure S1 - Automatic adjustment of source coordinates for tSSS.** A. Schematic representation of the decomposition of the magnetic field perceived at the sensor level into three components originating from neural and artefactual sources. Image reproduced from the Elekta Neuromag *MaxFilter^TM^* User’s Guide (Elekta Neuromag Oy, Helsinki, Finland). B. Example of optimal (left) and suboptimal (center) subject’s head positioning in the MEG scanner. Right: schematic representation of the automatic selection of the center of the sphere of interest, *S_in_*, as the midpoint of the segment linking the head sphere and the sensor sphere centers. The head sphere is represented in green, solid trace; the sensor array sphere is represented in blue; the sphere of interest is represented in orange. The dotted green line represents the sphere of interest that one would obtain when setting its center equal to the head sphere center. C, D. Data from two example subjects, with optimal (C) and suboptimal (D) head position in the MEG helmet. The insets represent the head positions inside the helmet. The graphs represent the source-signal average power content and the average power spectrum for different tSSS preprocessing settings, i.e., when moving the center of *S_in_* center from the *S_head_* center (in green) towards the sensors’ array, passing by the *S_sensors_* center (in blue). Bars indicate mean and standard deviation values over recorded epochs of duration 3.3 s. The green, orange and blue bars indicate the average power contents obtained when setting *S_in_* equal to the *S_head_* center; when automatically setting the *S_in_* center as described above; when setting *S_in_* equal to the *S_sensor_* center, respectively. The shaded areas in the graphs localize the head (green), the optimal sphere center area (orange), and the area between the *S_sensors_* center and the sensors’ array, i.e., very close to the sensors’ array (grey). In the power spectra, the green/orange/blue lines represent the spectra obtained when setting *S_in_* center equal to the *S_head_* center, to the automatically selected center (*S_optimal_*), and to the *S_sensor_* center, respectively. The spectra obtained when moving the *S_in_* center from the *S_head_* center towards the sensors’ array are displayed in progressively lighter shades of grey.


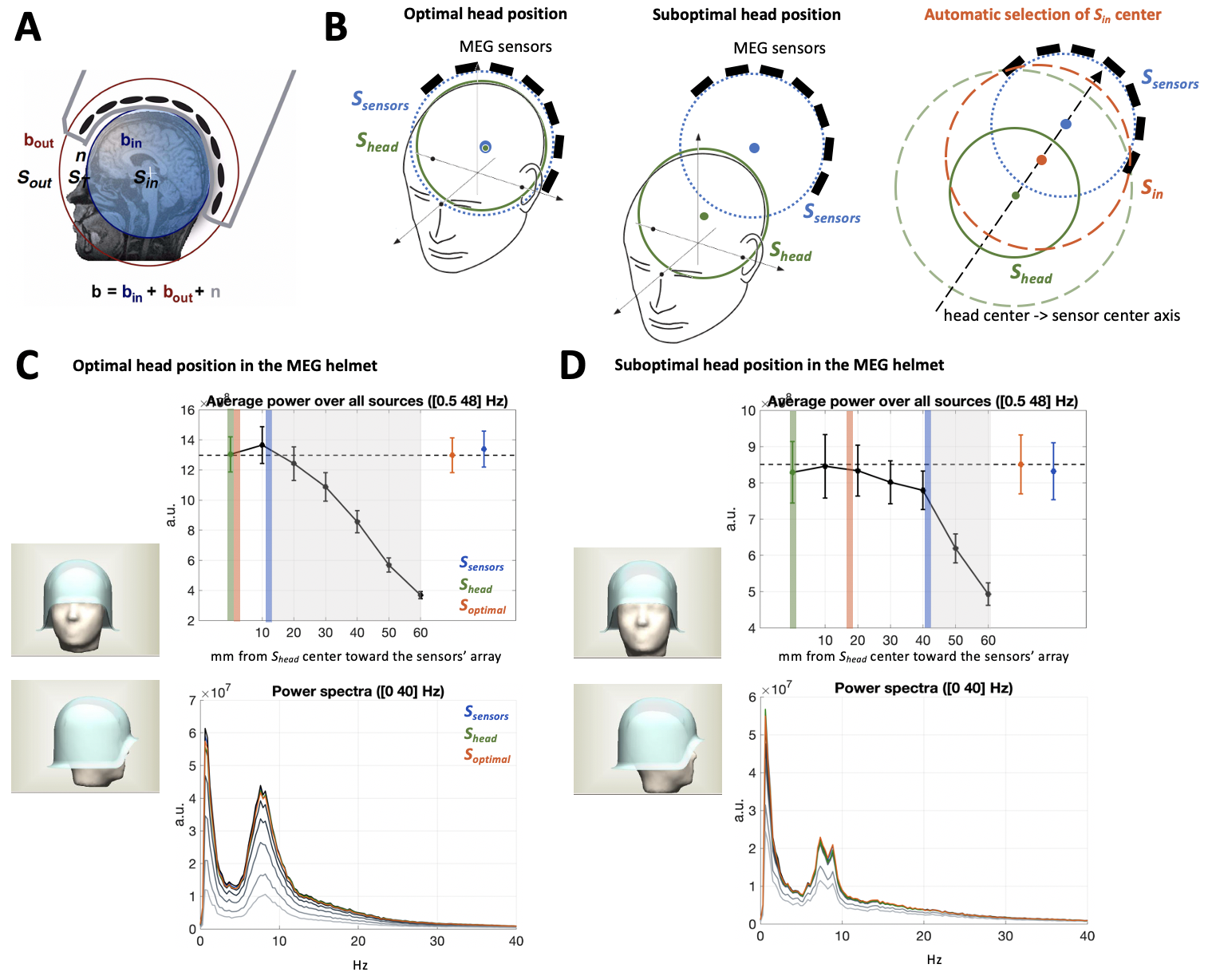


**Figure S2 – No relationship between head position and spectral features.** Scatter plots representing the individual peak frequency (IPF) (left), relative alpha1-power content over the occipital sources (center), and global power (average over all frequency bands and sources; right) as a function of the distance between the head sphere (*S_head_*) and sensor array sphere (*S_sensors_*) centers. Each dot represents a single subject; frequency-domain measures were averaged over 8 epochs of 13.1 seconds duration. Pearson’s correlation (*r*) and p-values (*p*) are reported above each plot.


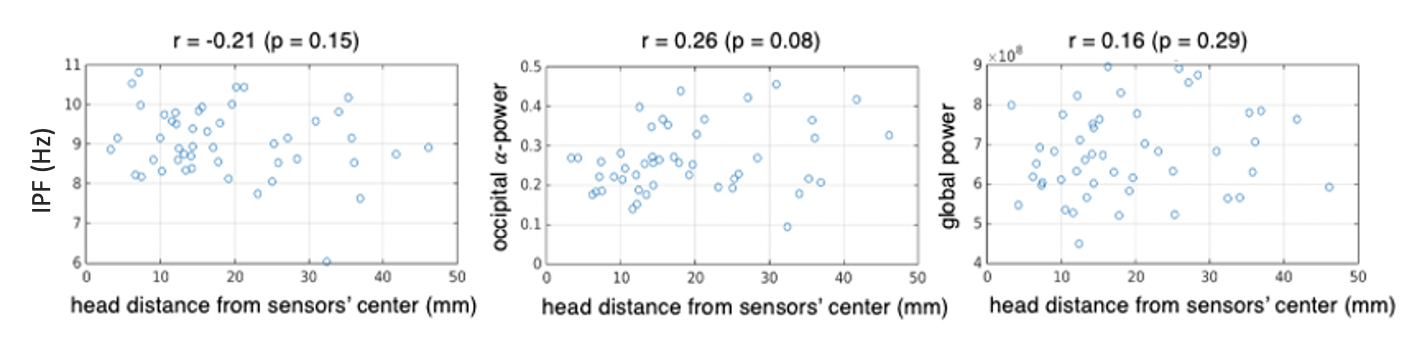


**Figure S3 – Epoch selection.** The automatic epoch selection for individual subjects was based on the skewness of the time series amplitude distributions (an indicator of presence of artefacts), individual peak frequency and occipital alpha1 relative power content (possible indicators of subject drowsiness). For an example subject: A. Relative power content in the alpha1 band (8-10 Hz) for different epochs and cortical regions. The occipital sources in the left and right hemisphere correspond to the two vertical stripes with higher power contents. B. Individual peak frequencies (spectral peak in the range 4-13 Hz) for different epochs. The continuous and dotted lines indicate the mean value ± 1.282 standard deviations, respectively. C. Epoch ranking based on the occipital alpha1 relative power content and individual peak frequency. To assess the epoch ranking with respect to these two parameters, both the individual peak frequency and the occipital alpha1 power content were rescaled (normalized) to the range 0-1. Epochs selected for further analyses for this example subject are represented in orange.


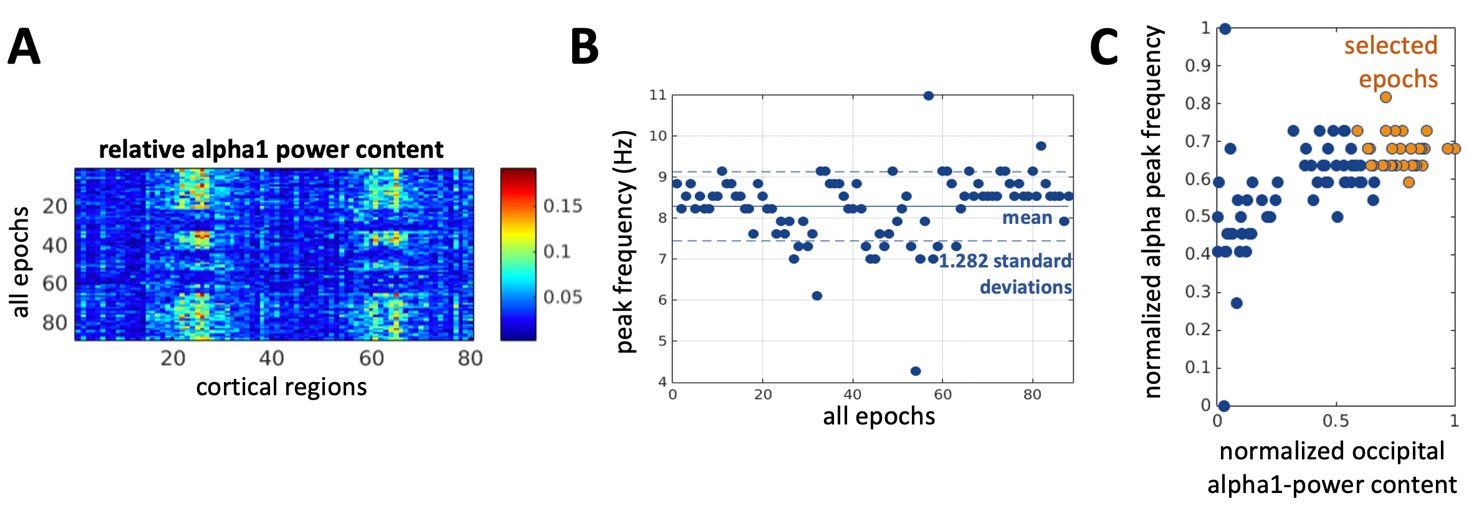


**Figure S4 – Group-average functional connectivity matrices.** Group-average functional connectivity matrices for the 6 considered frequency bands (delta (0.5-4 Hz), theta (4-8 Hz), alpha1 (8-10 Hz), alpha2 (10-13 Hz), beta (13-30 Hz), gamma (30-48 Hz) band), for (A) cognitively normal and (B) cognitively impaired participants. Functional connectivity between every pair of cortical regions was quantified with the Amplitude Envelop Correlation measure after pairwise orthogonalization of the original time series.


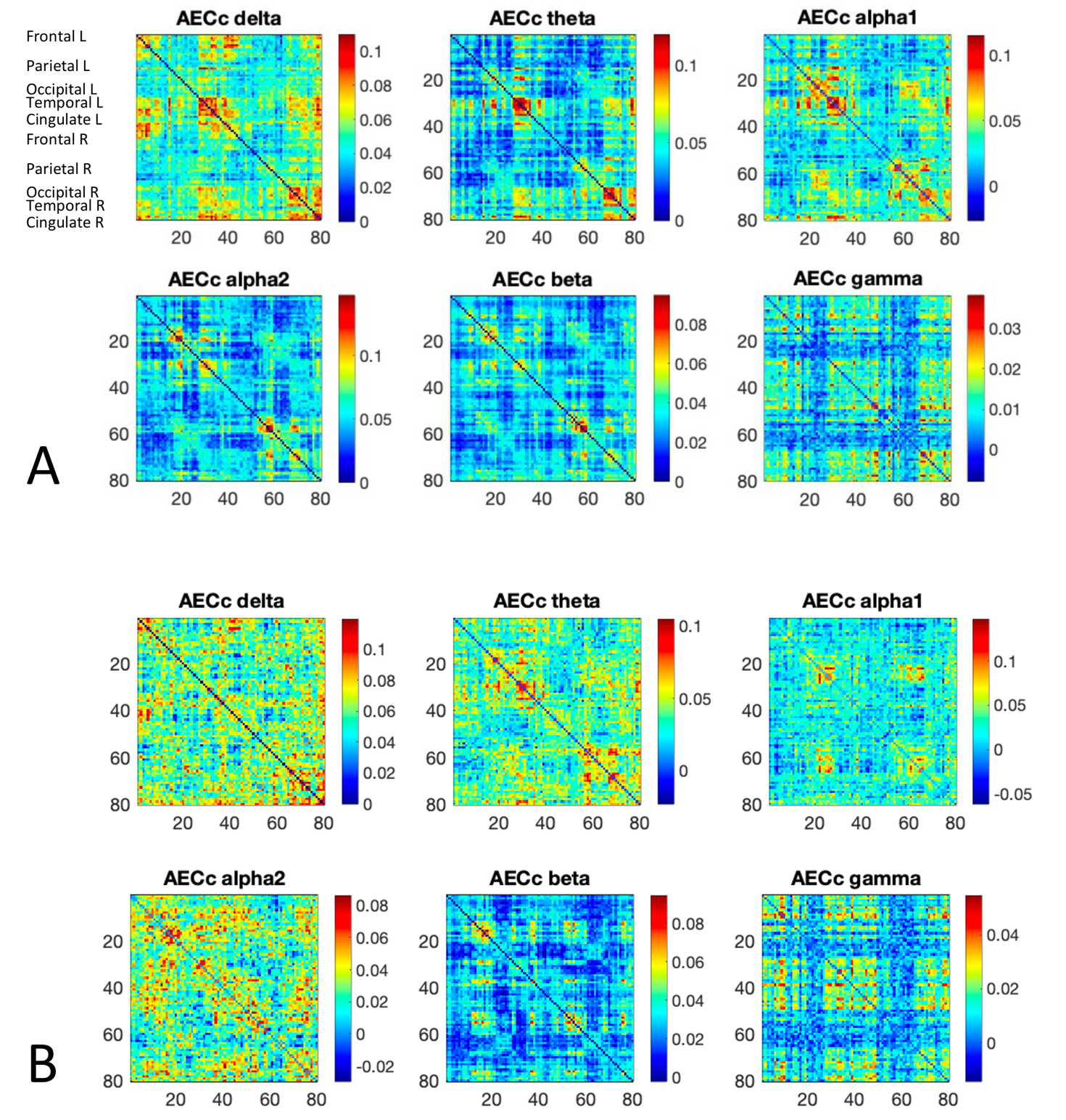


**Figure S5 – Multivariate correlation patterns between electrophysiological and cognitive features evaluated in 35 cognitively normal oldest-old subjects.** Each panel represents, from left to right: (i) the brain loadings, (ii) the cognitive loadings, and (iii) the data projection onto the brain and cognitive saliences for the first (A) and second (B) significant multivariate correlation patterns between spectral and cognitive features, and for the significant multivariate pattern between functional connectivity and cognitive features (C) from partial least square correlation (PLSC) analyses. In the loading plots, bars and dots represent the average and dispersion of brain and cognitive loadings over 500 bootstraps with replacement; loadings reliably different from zero are shaded in yellow. P-values for the multivariate correlation patterns are reported below the loading bar plots (* indicates pattern surviving multiple comparison correction at *FDR* < .05). In the scatter plots on the right, each dot represents brain and cognitive data of a single cognitively normal (CN) subject projected onto the corresponding PLSC saliences. The r-squared between the brain and cognitive data projection onto the PLSC saliences is reported above each scatter plot and quantifies the amount of cognitive scores’ variance explained by the spectral or functional connectivity features.

**
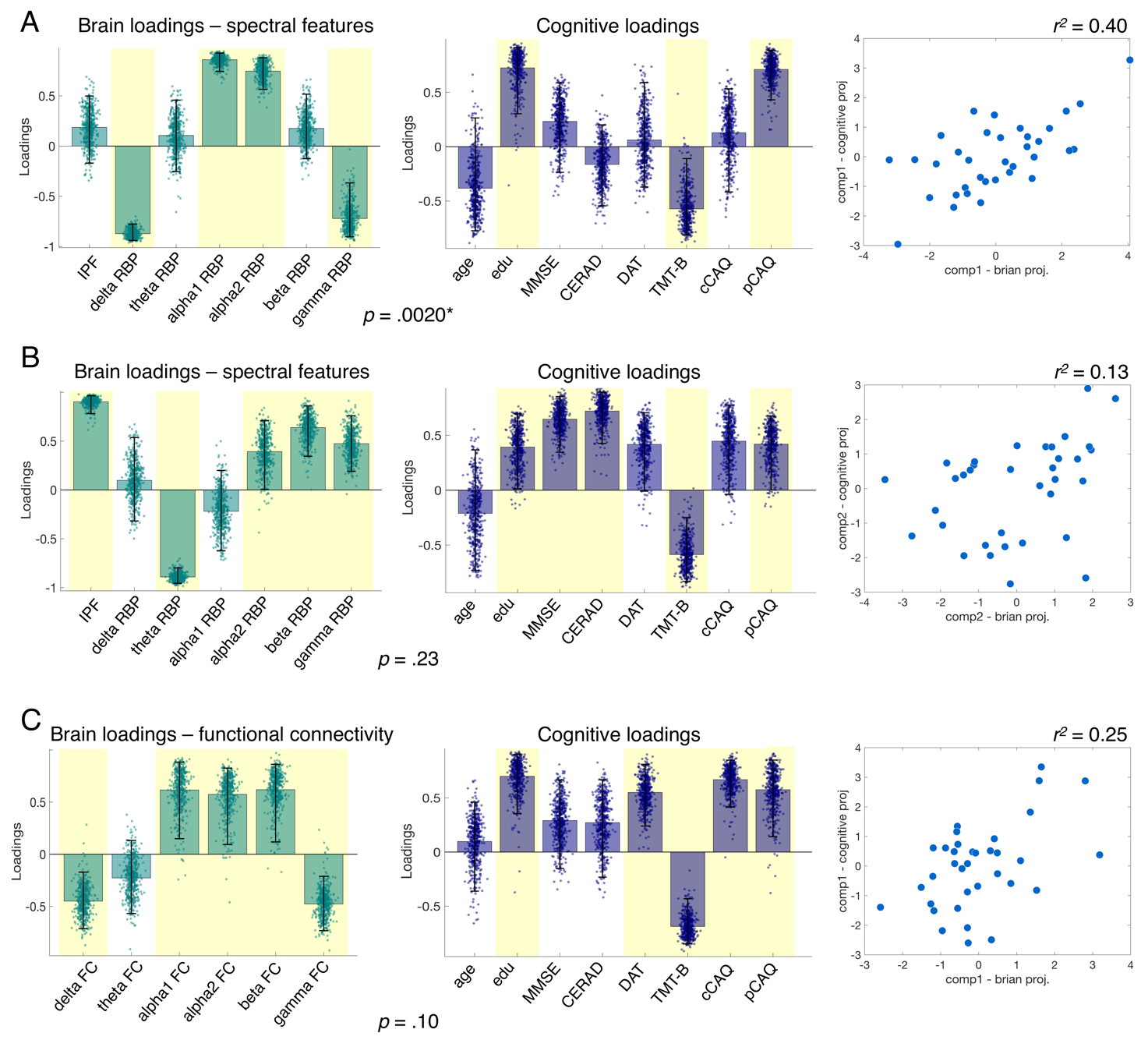
**

**References**

Gong, G., He, Y., Concha, L., Lebel, C., Gross, D. W., Evans, A. C., et al. (2009). Mapping Anatomical Connectivity Patterns of Human Cerebral Cortex Using In Vivo Diffusion Tensor Imaging Tractography. *Cereb. Cortex* 19, 524–536. doi:10.1093/cercor/bhn102.

Hari, R., and Puce, A. (2017). *MEG-EEG Primer*. Oxford, New York: Oxford University Press.

Taulu, S., and Hari, R. (2009). Removal of magnetoencephalographic artifacts with temporal signal-space separation: Demonstration with single-trial auditory-evoked responses. *Hum. Brain Mapp.* 30, 1524–1534. doi:10.1002/hbm.20627.

Taulu, S., Kajola, M., and Simola, J. (2004). Suppression of Interference and Artifacts by the Signal Space Separation Method. *Brain Topogr.* 16, 269–275. doi:10.1023/B:BRAT.0000032864.93890.f9.

Taulu, S., and Simola, J. (2006). Spatiotemporal signal space separation method for rejecting nearby interference in MEG measurements. *Phys. Med. Biol.* 51, 1759–1768. doi:10.1088/0031-9155/51/7/008.

Taulu, S., Simola, J., and Kajola, M. (2005). Applications of the signal space separation method. *IEEE Trans. Signal Process.* 53, 3359–3372. doi:10.1109/TSP.2005.853302.

Tzourio-Mazoyer, N., Landeau, B., Papathanassiou, D., Crivello, F., Etard, O., Delcroix, N., et al. (2002). Automated Anatomical Labeling of Activations in SPM Using a Macroscopic Anatomical Parcellation of the MNI MRI Single-Subject Brain. *NeuroImage* 15, 273–289. doi:10.1006/nimg.2001.0978.

Yeo, B. T., Krienen, F. M., Sepulcre, J., Sabuncu, M. R., Lashkari, D., Hollinshead, M., et al. (2011). The organization of the human cerebral cortex estimated by intrinsic functional connectivity. *J. Neurophysiol.* 106, 1125–1165. doi:10.1152/jn.00338.2011.
